# Serving patients in the COVID-19 pandemic

**Published:** 2020-09-01

**Authors:** Jagadesh C Reddy, Pravin K Vaddavalli

**Affiliations:** 1 Head: Cataract and Refractive Services, LV Prasad Eye Institute, Hyderabad, India.; 2 Director: The Cornea Institute, LV Prasad Eye Institute, Hyderabad, India.

**An Indian eye institute’s experience of coping with the COVID-19 pandemic.**

**Figure:**
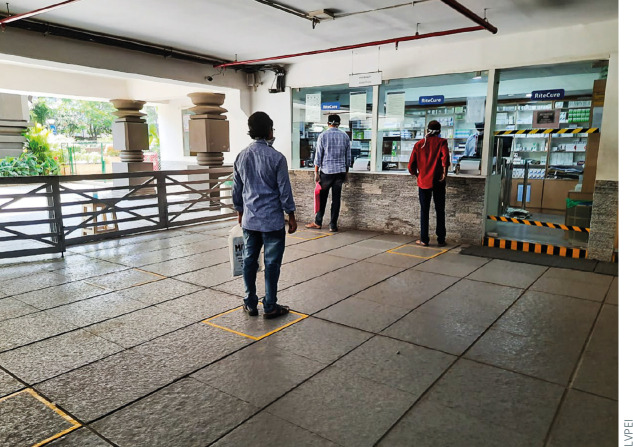
Markings on the floor facilitating adequate distancing between patients. **INDIA**

L V Prasad Eye Institute (LVPEI) has a network of 210 centres located in four states in south India. At the start of lockdown (24 March 2020) all the vision centres in the remote villages closed their doors to patients. The secondary and tertiary centres, and the centre of excellence, provided emergency care only.

Our approach to this pandemic was prioritised in the following way:

**Patient preparation.** We provided advice and reassurance to patients online in the form of posters (Figure 1) and a short video (**https://onlineapplicationform.lvpei.org/awareness-and-precautions/**), including, for each of the four states, phone numbers to call in case of an eye emergency.

**Safety.** Our centre for innovation came up with an open source design for a protective face shield, known as OS Visor, which can be made using a laser cutter. The design files can be downloaded from: **https://lvpmitra.com/osvisor**

**Patient flow.** We measured the temperature of all patients who walked into the centre and asked about their travel history and respiratory symptoms. Patients who did not have any symptoms indicating an eye emergency were politely requested to come back at a later date.

**Triage team.** The triage team consisted of an ophthalmologist (in a glass cabin and wearing personal protective equipment), who would determine whether the patient posed a risk of spreading COVID-19 and assess the need for an immediate eye examination.

**Teams.** The entire patient care service was divided into three teams, each consisting of ophthalmologists, receptionist, counsellors, an administrator, a pharmacist and telecounsellors. Each team visited the hospital every third day. Staff members never changed teams, thereby avoiding cross-infection.

**Figure 1:**
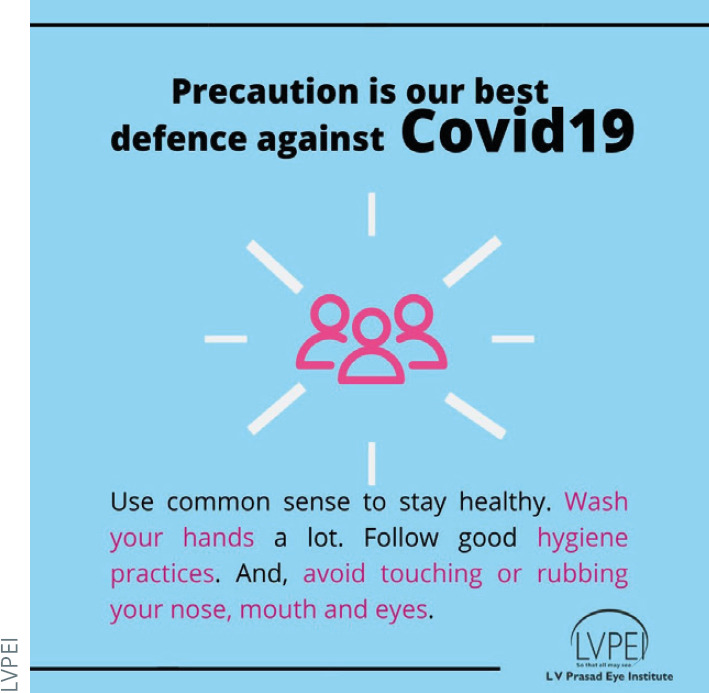
One of the patient awareness posters available on the LVPEI website

**Figure 2:**
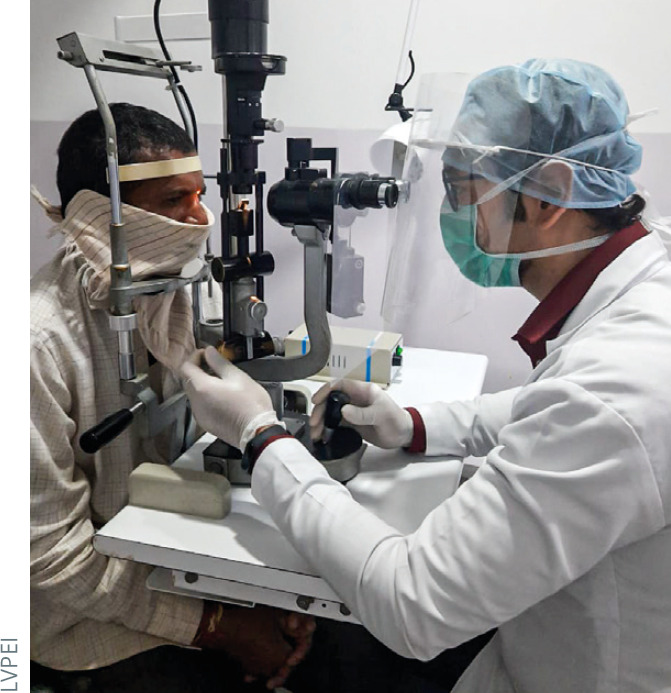
Slit lamp examination by an ophthalmologist wearing the OS Visor

**Examination protocol.** We carried out critical examinations only. Interventions that increased proximity with the patient were avoided if possible. Each staff member used a face mask and visor, and patients used face masks (Figure 2).

## Services provided

- **Child eye health.** We continued our popular screening programme for retinopathy of prematurity and provided all necessary treatments, such as lasers, intravitreal anti-VEGF injections and vitreoretinal surgery. Children with a new diagnosis of retinoblastoma, or who required a follow-up examination for retinoblastoma, were examined and treated.
- **Emergency care.** We treated everyone with acute trauma and infection. The most common procedures were repairing of open globe injuries, application of tissue adhesive for perforated corneal ulcers, vitreous biopsy and intraocular antibiotic injections for endophthalmitis, retinal detachment procedures, intravitreal injections for acute retinitis and age-related macular degeneration, and cataract extraction for lens- induced glaucoma.
- **Teleophthalmology.** Clinicians proactively made phone calls to all the patients whose appointments were cancelled and provided advice over the phone. Secure access to patients’ electronic medical record (EMR) was provided to all clinicians via desktop sharing software.

## Future plans

When the curve for COVID-19 flattens, we will reopen services in a staged manner, with special emphasis on the following:

- Availability of protective gear for all staff members
- Availability of consumables and medical supplies
- Clarity on number of patients to be seen in each hour
- Measures to prevent overcrowding in the waiting areas
- Making staff members available in teams
- Making available protocols for patients and staff to follow so they can remain safe through the process.

